# Supplementary material for: Ultrafast excited state dynamics and light-switching of [Ru(phen)2(dppz)]2+ in G-quadruplex DNA
Source: Commun Chem. 2021 May 14;4:68. doi: 10.1038/s42004-021-00507-0 (PMC9814642; doi:10.1038/s42004-021-00507-0)
Supplement: Supplementary file 1 — Supplementary Information [file 42004_2021_507_MOESM1_ESM.pdf]

Supplementary Material

Ultrafast excited state dynamics and light-switching of $[Ru(phen)_2(dppz)]^{2+}$ in G-quadruplex DNA

Chunfan Yang,[1] Qian Zhou,[1] Zeqing Jiao, [1] Hongmei Zhao, [2] Chun-Hua Huang, [3] Ben-Zhan Zhu, [3]and Hongmei Su[1,*]

1.College of Chemistry, Beijing Normal University Institution No.19, Xinjiekouwai St, Haidian District, Beijing, 100875, China

E-mail: hongmei@bnu.edu.cn

2. Institute of Chemistry, Chinese Academy of Sciences, Zhongguancun North First Street 2,100190 Beijing, China

3. State Key Lab of Environmental Chemistry and Ecotoxicology, Research Center for Eco-Environmental Science Chinese Academy of Sciences, Beijing 100085, China

CD spectra of G-quadruplexes

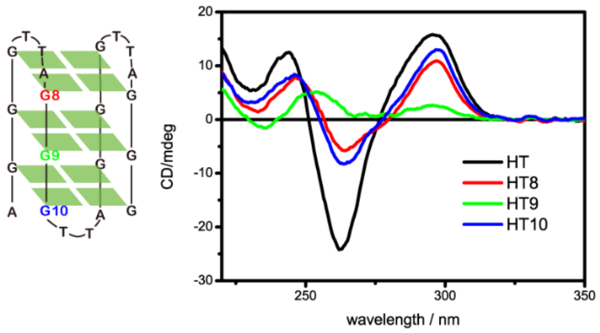

Supplementary Figure.1. CD spectra of normal $AG_3(T_2AG_3)_3$ and the mismatch G-quadruplex: HT8, HT9 and HT10, with the thymine (T) being substituted by G base in the position of G8, G9 and G10, respectively.

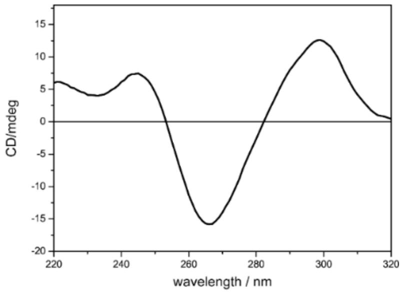

Supplementary Figure.2. CD spectra of *Oxyticha nova* G-quadruplex

Supplementary Methods

Melting curves

Melting curves were collected by CD spectra as a function of temperature. The temperature of the solution was increased from 30 to 90 °C at a rate of 0.5°C/min, and the absorbance at 295 nm was continuously monitored for solutions of G quadruplex DNA in the absence and presence of the $[Ru(phen)_2dppz]^{2+}$.

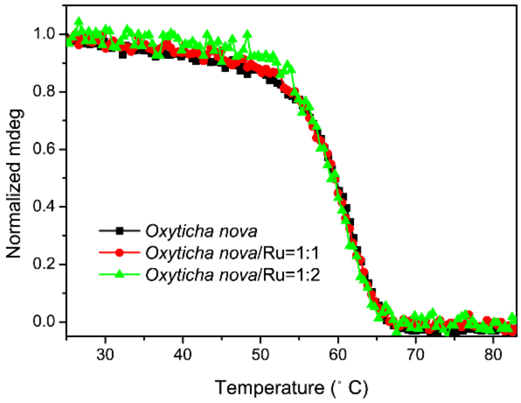

Supplementary Figure.3. Normalized CD melting curves for *Oxyticha nova* G-quadruplex in the absence and presence of $[Ru(phen)_2dppz]^{2+}$. The stability of G-quadruplexes DNA was assessed by CD signal at 295 nm.

Absorption spectra titration

Absorption spectra titrations were carried out at room temperature to determine the binding constant between DNA and complex. Initially, 2000 μL solutions of the buffer and the ruthenium complex sample (~13 μM) were placed in the reference and sample cuvettes (1.0 cm path length), respectively. During the titration, aliquot (1-10 μL) of buffered DNA solution was added to each cuvette to eliminate the absorbance of DNA itself, and the solutions were mixed by repeated inversion. After the solutions were mixed for ~5 minutes, the absorption spectra were recorded. The titration processes were repeated until there was no change in the spectra, indicating binding saturation had been achieved. The intrinsic binding constants *K* with each DNA at 25 °C were obtained using the following equation, [1-2]

$$(\varepsilon_a - \varepsilon_f) / (\varepsilon_b - \varepsilon_f) = (b - \left(b^2 - \frac{2K^2 C_t [DNA]}{s}\right)^{\frac{1}{2}} / 2KC_t$$

$$b = 1 + KC_t + K[DNA]/2s$$

where *[DNA]* is the DNA concentration in base pair, ε_a, ε_f and ε_b are, the apparent extinction coefficient (A/[M], or A/ C_t), the extinction coefficient for free metal complex (M) and the extinction coefficient for the metal complex (M) in the fully bound form respectively. Here, ε_b is determined when the concentration ratio [G-quadruplex]/[M] = 4:1. *K* is the equilibrium binding constant in M^{-1}, C_t is the total metal complex concentration, and *s* is the binding site size (in base pairs) of the small molecule interacting with DNA.

Supplementary Table 1. Obtained binding constant *K* and binding size *s*, according to the above equations.

	K/ M^{-1}	*s*
HT	5.1×10^6	2.3
Oxyticha nova	0.99×10^6	1

Emission spectra titrations

Emission spectra were measured on a HITACHI F-4600 Spectro fluorophotometer. The excitation wavelength was 439 nm, and the emission spectrum was collected from 550 to 800 nm. Excitation and emission slits were set at 5 and 5 nm, respectively. A 2000 μL solution of 10 μM $[Ru(phen)_2dppz]^{2+}$ was kept in a 1.0 cm path length quartz cuvette. 1-10 μL of DNA solution was then added to the sample cell. After mixed 5 minutes, the spectrum was taken. The titration processes were repeated until there were no changes of emission intensities.

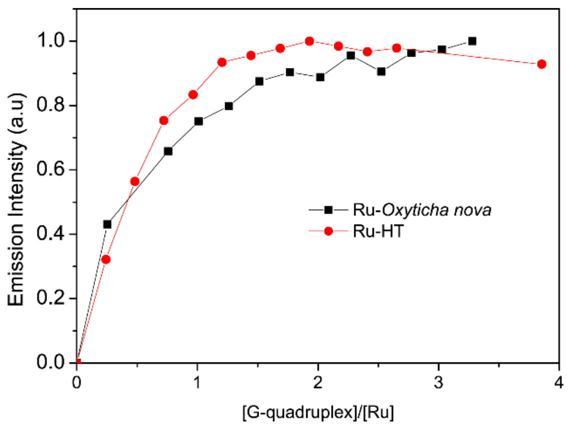

Supplementary Figure.4. Fluorescence intensity of [Ru(phen)$_2$dppz]$^{2+}$ at 620 nm in a titration with HT (red) and *Oxyticha nova* (black) G-quadruplex.

Steady-state and transient measurement results for Oxyticha nova G-quadruplex

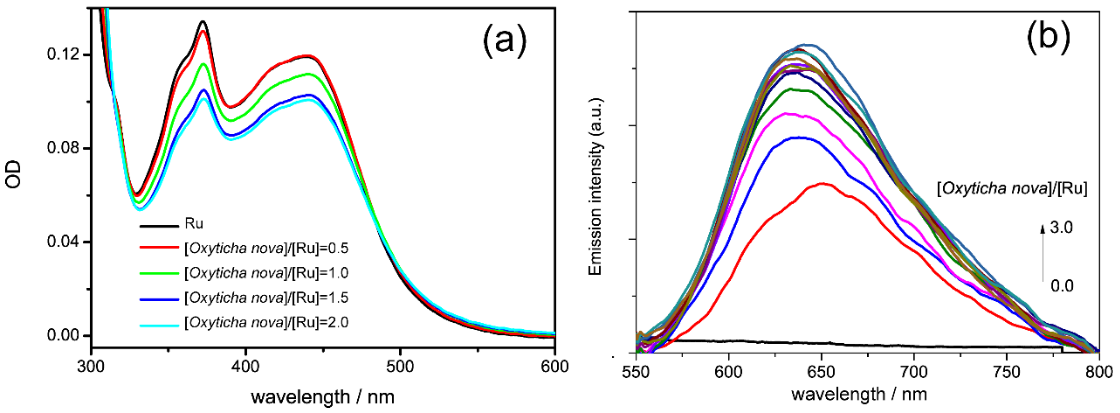

Supplementary Figure.5. (a) UV-vis absorption spectra of 5 μM [Ru(phen)$_2$dppz]$^{2+}$ alone and when bound to *Oxyticha nova* G-quadruplex at several concentration ratios. (b) The emission spectra of [Ru(phen)$_2$dppz]$^{2+}$ alone and when bound to *Oxyticha nova* G-quadruplex at several concentration ratios.

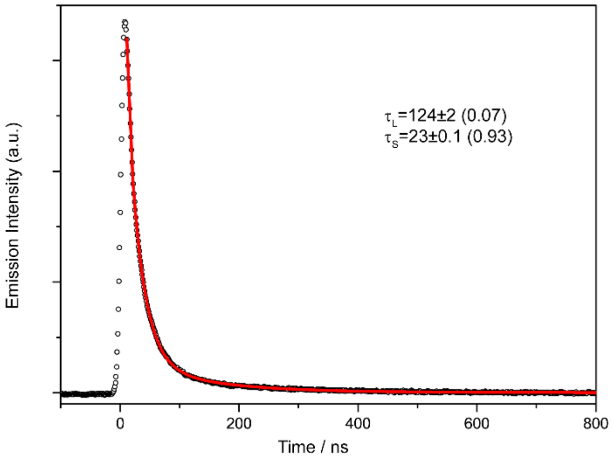

Supplementary Figure.6. Experimental (black) and fitted (red) decay dynamics curves of ^{3}MLCT luminescence at 620 nm for [Ru (phen)2dppz]2+ when bound to Oxyticha nova G-quadruplex in 10 mM Tris-HCl, and 100 mM NaCl buffer (pH 7.5) upon 355 nm excitation. Bi-exponential functions are applied to obtained the time constants shown in the figure.

The emission intensities of $[Ru(phen)_2dppz]^{2+}$

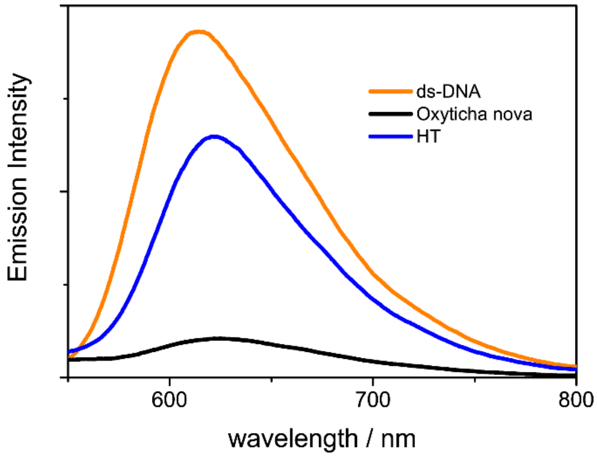

Supplementary Figure.7. The emission spectra of $[Ru(phen)_2dppz]^{2+}$ when bound to ds-DNA, HT and *Oxyticha nova* G-quadruplex at ratios 1:2.

When $[Ru(phen)_2dppz]^{2+}$is bound to ds-DNA, HT and Oxyticha nova, respectively, the steady state emission intensities follow the order: ds-DNA>HT> Oxyticha nova, which is consistent with the the time-resolved luminescence decay kinetics data. In contrast to the intercalation binding mode in ds-DNA, the stacking binding mode with quartets in G-quadruplex may provide less protection for the Ru complex from water quenching. Therefore, the emission is the strongest when bound to ds-DNA. Moreover, the bilateral TTA loop region of HT and the TTTT diagonal loop region of Oxyticha nova provide different protections for the Ru complex, and the binding constants for these two G-quadruplex are different (Supplementary Table 1: HT> Oxyticha nova), so the emission intensities of the Ru complex in HT is larger than Oxyticha nova.

For the mismatch G-quadruplexes in comparison to natural HT as shown in Figure 2(b), the emission intensity of the Ru complex follows the order: HT8> HT~HT10, consistent with the the time-resolved luminescence decay kinetics data (Table 1). This agree with our discussion in the paper: 1) replacement of T base (mismatch) tends to weaken the π-π stacking of G-quartets and such changes should be helpful for the ruthenium complexes approaching G-quadruplex core, and can render phenazine nitrogens relatively well shielded by the quartet and loops:2) $[Ru(phen)_2dppz]^{2+}$ mainly stacks on the terminal of the G8-quartet with the lateral loops. The replacement of G10 by T doesn't lead to obviously different emission intensity in HT10. So, the ruthenium bound to HT8 has the strongest emission intensity.

Femtosecond time-resolved transient absorption spectroscopy

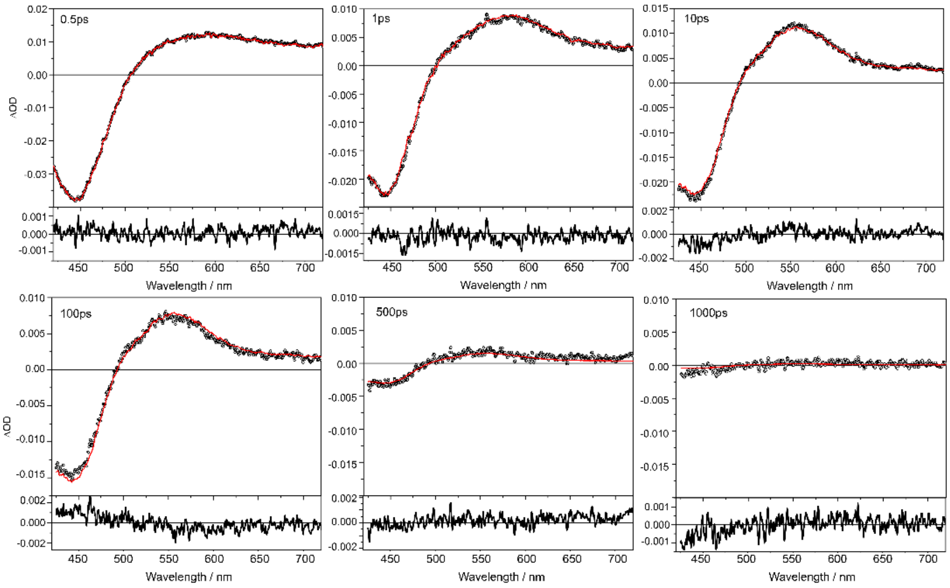

Supplementary Figure.8. Ultrafast transient absorption spectra of $[Ru(phen)_2dppz]^{2+}$ upon 400 nm fs laser excitation in water. The concentrations used are $[Ru(phen)_2(dppz)]^{2+}$=66 μM. The fitting curves obtained from the global fit are shown with red lines.

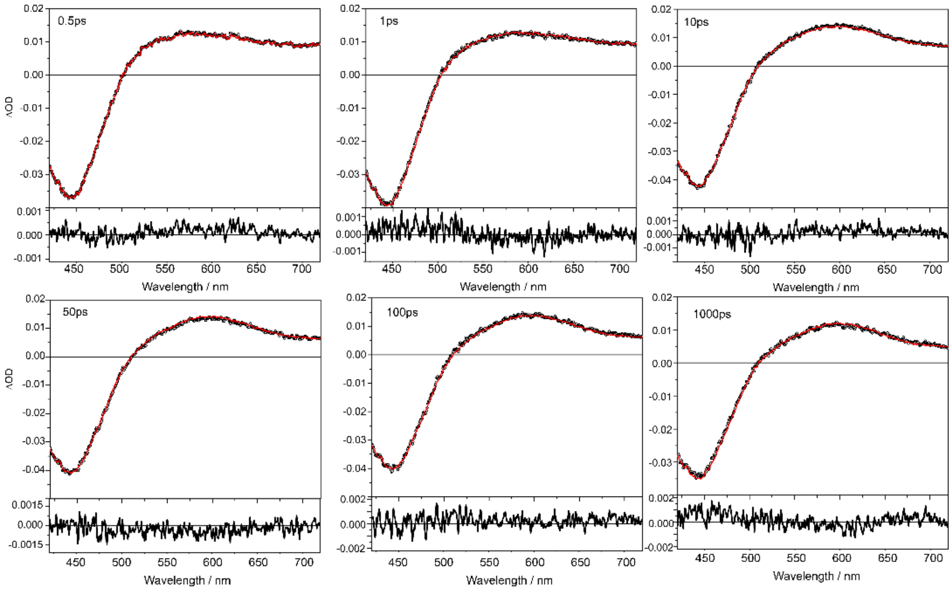

Supplementary Figure.9. Ultrafast transient absorption spectra of $[Ru(phen)_2dppz]^{2+}$ and HT G-quadruplex upon 400 nm fs laser excitation in 10 mM Tris-HCl, and 100 mM NaCl buffer (pH 7.5). The concentrations used are $[Ru(phen)_2(dppz)]^{2+}$=66 μM, [DNA]=132 μM. The fitting curves obtained from the global fit are shown with red lines.

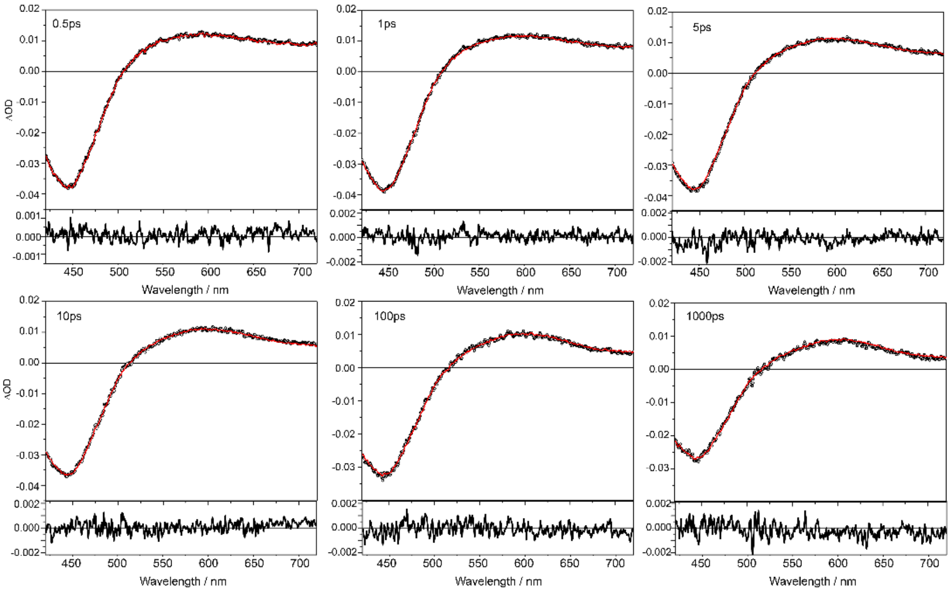

Supplementary Figure.10. Ultrafast transient absorption spectra of [Ru(phen)$_2$dppz]$^{2+}$ and *Oxyticha nova* G-quadruplex upon 400 nm fs laser excitation in 10 mM Tris-HCl, and 100 mM NaCl buffer (pH 7.5). The concentrations used are [Ru(phen)$_2$(dppz)]$^{2+}$=66 μM, [DNA]=132 μM. The fitting curves obtained from the global fit are shown with red lines.

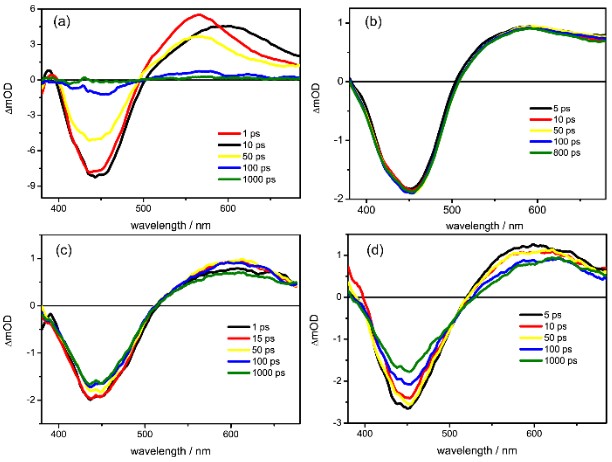

Supplementary Figure.11. Ultrafast transient absorption spectra of [Ru(phen)$_2$dppz]$^{2+}$ upon 350 nm fs laser excitation in water (a), ds-DNA (b), HT G-quadruplex (c), and Oxyticha nova G-quadruplex (d) in 10 mM Tris-HCl, and 100 mM NaCl buffer (pH 7.5). The concentrations used are [Ru(phen)2(dppz)]2+=66 μM, [DNA]=132 μM.

Supplementary References

[1] M. T. Carter, M. Rodriguez, A. J. Bard, *J. Am. Chem. Soc.* **1989**, *111*, 8901-8911.

[2] S. Shi, J. Zhao, X. Gao, C. Y. Lv, L. Yang, J. Hao, H. L. Huang, J. L. Yao, W. L. Sun, T. M. Yao, L. N. Ji, *Dalton Trans.* **2012**, *41*, 5789-5793.
